# How should professional psychiatric associations respond to a large-scale disaster?

**DOI:** 10.1192/j.eurpsy.2025.23

**Published:** 2025-02-19

**Authors:** Emre Mutlu, Alper Bülbül, Emre Cem Esen, İrem Ekmekçi Ertek, H. Rengin Güvenç, Koray Başar, M. Hamid Boztaş, M. İrem Yıldız, Münevver Hacıoğlu Yıldırım, Rümeysa Taşdelen, Ejder Akgün Yıldırım

**Affiliations:** 1 Department of Psychiatry, Hacettepe University, Ankara, Türkiye; 2 Department of Psychiatry, East London NHS Foundation Trust, London, UK; 3 Department of Psychiatry, İzmir University of Economics, İzmir, Türkiye; 4 Department of Psychiatry, Gazi University, Ankara, Türkiye; 5 Private Practice, İstanbul, Türkiye; 6 Department of Psychiatry, Bolu İzzet Baysal University, Bolu, Türkiye; 7 Department of Psychiatry, Prof. Mazhar Osman Mental Health Training and Research Hospital, İstanbul, Türkiye

**Keywords:** Disaster preparedness, earthquake, psychiatric associations, psychological first aid, psychosocial support

## Abstract

Disasters pose unique challenges, triggering significant psychological and social crises with both short- and long-term impacts. In this article, we address the critical role of professional psychiatric associations (PPAs) in responding to large-scale disasters, emphasizing the operational model connected with the Psychiatric Association of Türkiye’s (PAT) response to the 2023 earthquakes in Türkiye and Northern Syria. We propose the SOLIDARITE model, a structured response framework, which incorporates sustained preparedness, organized networks, resource libraries, on-site and remote interventions, and comprehensive disaster planning across early, middle, and long-term phases. The model emphasizes a multidimensional approach integrating pre-disaster preparedness through training, various psychosocial support options, the establishment of networks, and the formulation of a master disaster response plan. The implementation of this model by PAT during the 2023 earthquakes facilitated an effective and prompt response, underlining the importance of PPAs’ role in disaster preparedness and action. The SOLIDARITE model supports the need for deeper integration of disaster psychiatry into psychiatric training and calls for national and international collaboration to enhance the preparedness and response capacity of PPAs.

Mass trauma and disasters trigger significant psychological and social crises, both short- and long-term. In recent years, acute weather changes, often attributed to climate change, present as mass disasters and contribute significantly to the mental health burden associated with the change [1]. Short-term impacts of disasters include exacerbating preexisting conditions, including severe mental disorders, along with emergency-induced problems predisposing to mental health issues such as family separation, loss, trauma, and poverty. Long-term effects often involve the persistence of short-term issues, secondary traumas, and healthcare service deterioration. While guidelines like the Inter-Agency Standing Committee offer basic principles for mental health and psychosocial support (MHPSS) [2, 3], they often lack cultural adaptability [3, 4]. While the majority of the principles may be followed by national and regional authorities, there is a wide variability in the level of preparedness of public institutions. Furthermore, following some major disasters, conventional response systems may be insufficient. Health professionals and their organizations can provide invaluable assistance in responding to disasters through their knowledge and experience. Concerning the role professional psychiatric associations (PPAs) can play, the majority of the guidelines exhibit a dearth of recommendations on strategies and coordination tailored to the capacity of professional organizations. This article aims to propose an operational model for PPAs’ response to large-scale disasters.

## Background

For decades following the 1999 earthquake, many experts have warned the public about the risk of a severe earthquake in the Marmara region, including İstanbul, the most populous city in Türkiye. In response to the ongoing risk, the Psychiatric Association of Türkiye (PAT) took proactive steps. Building on its earlier work on mass trauma, PAT established the Disaster Preparedness and Intervention Unit (DPIU) in July 2022. This unit was specifically designed to respond to natural or human-caused trauma. Six months later, on February 6, 2023, Türkiye and Northern Syria were hit by two powerful earthquakes, measuring 7.8 and 7.5 on the Richter scale. Many city centers were left inhabitable, with severe destruction of infrastructure, including first-response facilities, and more than 3 million people out of 11 million affected had to move to other cities. A year later, in the epicenter, Hatay is still in ruins. As mental health needs emerge from the hyperacute stage in disasters [5], PAT started its actions within hours of the first earthquake. PAT’s DPIU enabled an organized and prompt response to one of the biggest natural disasters of the 21st century [6].

## A response model based on needs, resources, and experience: SOLIDARITE

During disasters, several critical needs may arise that a PPA could address. Predisaster actions include ensuring sustained preparedness, organizing internal and external networks, and establishing a comprehensive disaster master plan. Following a disaster, rapid response actions such as regional assessment, on-site interventions, and remote support become crucial. Additionally, PPAs should develop a robust library of resources and provide ongoing training and supervision to mental health workers to prepare for potential disasters.

**Figure 1.**
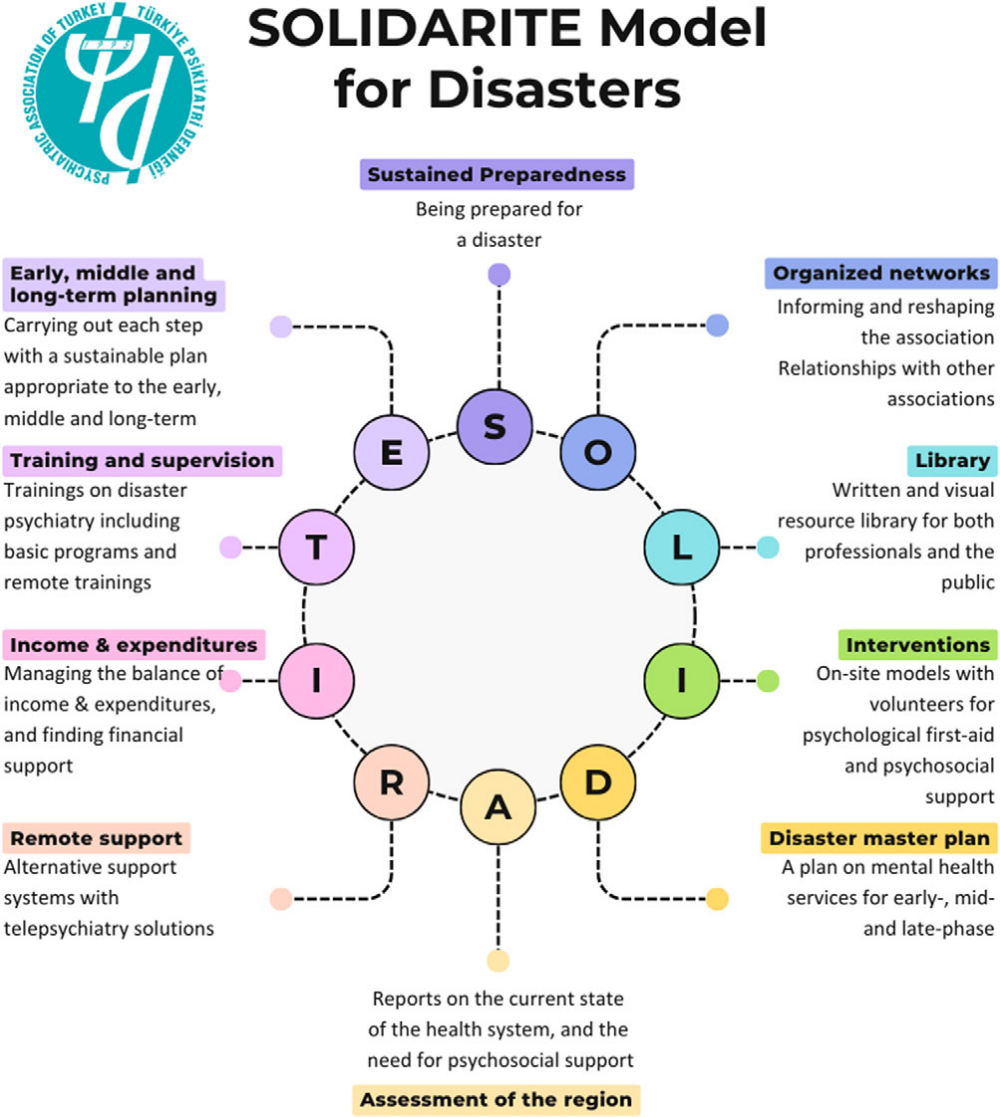
Description of the SOLIDARITE model, focusing on professional psychiatric associations for an organized response to a disaster.

The proposed **SOLIDARITE** model, illustrated in Figure 1, outlines key actions that PPAs should consider. The model includes 10 key steps: (1) sustained preparedness, (2) organized internal and external networks, (3) library of resources, (4) interventions on-site, (5) disaster master plan, (6) assessment of the region, (7) remote support, (8) income and expenditures, (9) training and supervision, and (10) early, middle, and long-term planning. These steps are designed to address existing gaps in disaster psychiatry training, meet MHPSS needs, and fulfill organizational requirements. Each step will be further elaborated, drawing on the PAT’s experience during the February 6, 2023, earthquakes.

### Sustained preparedness

If possible, a specialized section or unit will facilitate preparations. The DPIU established by the PAT 6 months before the earthquakes made organizing the association and training modules specified for disaster and trauma-related disorders easier. The DPIU comprised 10 members, including the Section on Trauma and Disaster Psychiatry chairs, with subunits responsible for volunteer actions, training, international cooperation, and financial support. With or without instituting a specialized unit, books, publications, training, and continuing medical education (CME) activities related to disaster response contribute to preparedness.

### Organized internal and external networks

Reshaping the organization flexibly, including the committees, branches, and sections according to emerging needs, is essential. PAT’s response was managed by the Disaster Crisis Management Committee formed by the Executive Committee, DPIU, and members from regions as representatives [7]. Furthermore, collaborating with local administration and other mental health organizations, such as professional associations of psychologists, social workers, and public health specialists, becomes beneficial. “Psychosocial Solidarity Networks,” which were formed and functioned effectively in earlier human-caused mass traumas, were reestablished in three major cities [6]. Since relocating to other cities is common in mass disasters, these networks and branch organizations are crucial in nonaffected areas. Pairing branches with each other can be advantageous in preparing for possible disasters in large geographical areas.

### Library of resources

PAT created a web page called “Earthquake and Mental Health,” which quickly expanded to include documents, video recordings, and pamphlets from various PAT sections [8]. Pamphlets containing information and practical recommendations on mental health first aid, which are more practical to distribute in fields lacking or deficient in internet or electricity, were prepared in the three most spoken languages in the area and distributed. Also, live broadcasts and podcasts on social and mainstream media can convey content to the general public. PAT used its weekly online program, “From Psychiatry to the Agenda.”

### Interventions on-site

On-site models with volunteers for psychological first aid and psychosocial support are needed when conventional health facilities are insufficient. After PAT’s calls, nearly 900 members applied as volunteers. In 8 months, 150 members served as volunteers to organize and provide mental health services in four local stations installed in mostly affected centers [9]. The logistical needs had to be covered by PAT.

### Disaster master plan

Preparations and the early response should be guided by a well-designed “Disaster Master Plan,” which must be developed by health authorities and should be open to modifications to ensure effective and sustainable actions. The master plan should be carefully developed, considering every aspect of the disaster, local risks, and capabilities. PPAs, professional, and nongovernmental organizations should play an advisory role in developing the disaster master plan. PAT has been working on a comprehensive master plan for mental health services for an expected earthquake in İstanbul.

### Assessment of the region

This step requires the exploration of the current state of the health system, the organization of mental health services, and the need for MHPPS to identify urgent requirements and implement activities safely. It is also important not to overlook the needs of psychiatrists in the region and the chronically ill who may be left unattended. PAT has been noted as the first organization to release an assessment report in the aftermath of the recent earthquakes [6]. Subsequent assessments of the region were conducted in the second week, the first month, and the sixth month.

### Remote support

Telehealth applications offer promising opportunities. PAT established an “Online Support System” for mental health problems, using video conferencing to offer psychological first aid and support to earthquake survivors, healthcare workers, and first responders. Seventy-four volunteering psychiatrists contributed to the system in the first 4 months, during which nearly 800 sessions were conducted [10]. The system could receive applications 12 h a day, 7 days a week, and involved collaboration with field volunteers and the local healthcare system through a workflow algorithm. Additionally, PAT applied hotlines or phone calls during the COVID-19 pandemic and the 2020 İzmir Earthquake.

### Income and expenditures

Finding financial support, balancing income and expenditures, and using the budget effectively were all challenges for a PPA. To address this, the PAT established the Finance Committee to ensure that donations and funds devoted to earthquake activities were obtained timely and projects were managed appropriately. On behalf of PAT, we express our sincere gratitude to those who supported our response.

### Training and supervision

Disaster psychiatry is not a core component of psychiatry training in all countries, yet the need for healthcare workers equipped with the necessary skills is critical during disasters. Basic training should be considered as preparedness for disasters, including remote training options in urgent situations. PAT organized a webinar series in the first week of the earthquakes, offering 4 days of lectures on preventive and therapeutic mental health care [6]. These lectures, attended by up to 2,500 participants, were later published as a guidance paper [11]. Weekly online supervision and Q&A sessions followed the webinars. Later, group interventions and supervision were conducted for the Ministry of Health and the Ministry of Family and Social Services staff in the earthquake area. In addition, institutions may be affected and residency programs may be interrupted to different degrees. Residency programs should be supported with complementary training in a large-scale disaster by PPAs.

### Early, middle, and long-term planning

Each step should be considered within a three-phased plan. As the needs and resources change in the aftermath of a disaster, it should be ensured that the response is carried out with a sustainable plan appropriate to the early, middle, and long-term periods.

Each of these steps is interconnected. For instance, on-site or remote interventions are only effective with a thorough regional assessment. Furthermore, ignoring the balance of income and expenditures could hinder the activities outlined in the master plan. Although managing all these elements may seem challenging, PPAs must play a pivotal role in policy-making, planning, and organizing disaster responses. Their involvement, support, and sometimes leadership may be crucial in ensuring a coordinated and effective response.

The SOLIDARITE model has significant challenges and limitations. A national psychiatric association cannot and should not be the sole responsible for delivering all mental health and psychosocial services. Major challenges included logistical costs and maintaining volunteer support, considering several risks related to volunteerism, such as inexperience, traveling, and on-site safety threats. Collaboration with other mental health and medical associations, like the Turkish Medical Association, has been essential in overcoming some obstacles.

Assessing the disaster’s aftermath and the response offers crucial insights for the future. National psychiatric associations should adopt a proactive policy for disaster preparedness, playing a key role in shaping well-designed master plans for disaster responses. Flexibility in organizational structure is essential to ensure efficient and timely reactions. Additionally, there must be a stronger focus on integrating disaster psychiatry into residency programs and continuing professional development. Leading efforts to build a network of solidarity, both nationally and internationally, is equally vital. In line with this framework, we recommend sharing experiences, discussing the evolving role of PPAs in disasters, collaborating with countries experienced in disaster psychiatry, and fostering a new approach to establishing a SOLIDARITE network for disaster preparedness and response.

## Data Availability

Not applicable.
